# The Effect of Palmitoylethanolamide (PEA) on Skeletal Muscle Hypertrophy, Strength, and Power in Response to Resistance Training in Healthy Active Adults: A Double-Blind Randomized Control Trial

**DOI:** 10.1186/s40798-024-00732-6

**Published:** 2024-06-07

**Authors:** Zoya Huschtscha, Jessica Silver, Michael Gerhardy, Charles S. Urwin, Nathan Kenney, Viet Hung Le, Jackson J. Fyfe, Simon A. Feros, Andrew C. Betik, Christopher S. Shaw, Luana C. Main, Gavin Abbott, Sze-Yen Tan, Anthony May, Craig M. Smith, Vicky Kuriel, Jackson Barnard, D. Lee Hamilton

**Affiliations:** 1 School of Exercise and Nutrition Sciences, Institute for Physical Activity and Nutrition (IPAN), Deakin University, Geelong, 3216 Australia; 2 Centre for Sport Research (CSR), School of Exercise and Nutrition Sciences, Deakin University, Geelong, 3216 Australia; 3 School of Exercise and Nutrition Sciences, Deakin University, Geelong, 3216 Australia; 4 School of Medicine, Institute for Mental and Physical Health and Clinical Translation (IMPACT), Deakin University, Geelong, 3216 Australia; 5 Respiratory Research@Alfred, Monash University, Melbourne, Australia

**Keywords:** Palmitoylethanolamide, Pain, Hypertrophy, Strength, Leg strength, Countermovement jump, Recreationally active

## Abstract

**Background:**

Palmitoylethanolamide (PEA) has analgesic/anti-inflammatory properties that may be a suitable alternative to over-the-counter (OTC) non-steroidal analgesics/anti-inflammatories. While OTC pain medications can impair strength training adaptations, the mechanism of action of PEA is distinct from these and it may not negatively affect skeletal muscle adaptations to strength training.

**Methods:**

The primary aim of this study was to investigate the effects of daily PEA supplementation (350 mg Levagen + equivalent to 300 mg PEA) combined with 8-weeks of resistance training on lean body mass with secondary aims addressing strength, power, sleep, and wellbeing compared to placebo (PLA) in young, healthy, active adults. In a randomized, controlled, double-blinded trial, 52 untrained, recreationally active participants aged 18–35 y were allocated to either the PEA or PLA groups. Participants consumed either 2 × 175 mg Levagen + PEA or identically matched maltodextrin capsules during an 8-week period of whole-body resistance training. This trial assessed the pre- to post- changes in total and regional lean body mass, muscular strength (1-RM bench, isometric mid-thigh pull), muscular power [countermovement jump (CMJ), bench throw], pain associated with exercise training, sleep, and wellbeing compared with the PEA or PLA condition.

**Results:**

48 Participants were included in the final intention to treat (ITT) analysis and we also conducted per protocol (PP) analysis (n = 42). There were no significant between-group differences for total or regional lean muscle mass post-intervention. There was a significantly higher jump height (CMJ) at week 10 in the PEA group compared to the PLA (Adjusted mean difference [95% CI] *p*-value; ITT: − 2.94 cm [− 5.15, − 0.74] *p* = 0.010; PP: − 2.93 cm [− 5.31, − 0.55] *p* = 0.017). The PLA group had higher 1-RM bench press post-intervention compared with the PEA group (ITT: 2.24 kg [0.12, 4.37] *p* = 0.039; PP: 2.73 kg [0.40, 5.06] *p* = 0.023). No significant treatment effects were noted for any of the other outcomes.

**Conclusion:**

PEA supplementation, when combined with 8 weeks of strength training, did not impair lean mass gains and it resulted in significantly higher dynamic lower-body power when compared with the PLA condition.

*Trial Registration:* Australian New Zealand Clinical Trials Registry (ANZCTR: ACTRN12621001726842p).

**Supplementary Information:**

The online version contains supplementary material available at 10.1186/s40798-024-00732-6.

## Key Findings

- Palmitoylethanolamide (PEA) is a compound that works as an anti-inflammatory and analgesic for the management of pain that works on different biochemical pathways compared to other over-the-counter analgesics and non-steroidal anti-inflammatories.
- Our findings indicate that a daily dose of 300 mg of PEA administered during 8-weeks of strength training did not interfere with skeletal muscle hypertrophy gains and may improve lower body power (e.g., jump height) compared to the placebo group.
- PEA may fill the gap for athletes and the active population as an alternative method of pain management.

**Supplementary Information:**

The online version contains supplementary material available at 10.1186/s40798-024-00732-6.

## Background

Pain management for athletes is an important but often overlooked consideration. Athletes can experience pain from exercise training (e.g., delayed onset muscle soreness), acute or overuse injury, and a large fraction of female athletes experience menstrual pain, all of which can influence immediate and future performance [1]. A recent consensus statement highlighted that there is a lack of evidence-based guidelines for pain management in athletes and some researchers have noted a misuse of analgesics (e.g., paracetamol/acetaminophen) and non-steroidal anti-inflammatory drugs (NSAIDs) in both recreational, junior, and elite level athletes [2–4]. For instance, Olympic level athletes have a fourfold greater use of analgesics and NSAIDs than their age-matched non-active counterparts [5]. Aside from pain management, athletes reportedly take pain medications prophylactically for the prevention of pain, or to decrease recovery time following an injury [6, 7]. Additionally, pain can affect an athlete’s sleep quality which can lead to a decrease in recovery time and increase the athlete's susceptibility to injury, illness and decreased pain tolerance [8, 9].

However, despite the known effectiveness of these drugs for pain management, long term use of analgesics and NSAIDs can increase the risk of health problems such as peptic ulcer disease, acute renal failure, and in extreme cases stroke/myocardial infarction [5, 10, 11]. Additionally, over-the-counter (OTC) pain management agents are effective because they suppress inflammation and pain pathways by inhibiting cyclooxygenase-2 (COX-2), which prevents the conversion of prostaglandins such as arachidonic acid to pro-inflammatory proteinoids [12]. Proteinoids may have an important role in muscle tissue remodelling following exercise due to actions on anabolic signalling through the PI3K/Akt pathway [13, 14]. In the long term this may influence athletes’ overall recovery and athletic performance. As proof of principle, the COX-2 inhibiting drugs ibuprofen and acetaminophen both inhibit muscle protein synthesis following strength training in young healthy adults [15, 16]. However, intervention studies investigating the effects of NSAIDs during the course of a structured resistance training program have shown mixed results. Krentz et al. [17] found no significant differences in muscle hypertrophy or strength between those that consumed 400 mg of ibuprofen on training days compared to a placebo following 6-weeks of a structured resistance training program. In contrast, Lilja et al. [18] used a higher dose (1200 mg) of ibuprofen which reflects the dose commonly consumed by the athletic population and found that it significantly attenuated gains in quadriceps muscle mass compared to a low-dose provision of aspirin following 8 weeks of resistance training. One strength of the study by Lilja et al. [18] is the longer study period (8 weeks) that allowed for a better assessment of changes in skeletal muscle mass, when compared to the 6-weeks in the study by Krentz et al. [17]. However, one limitation of the study by Lilja et al. [18] is the lack of a placebo control group. Nonetheless, considering the importance of pain management for athletes’ overall performance, and the negative effects that regular use of COX-2 inhibitors may have on overall health and potential training adaptations, it would be beneficial to assess if alternative pain relievers interfere with training adaptations.

Palmitoylethanolamide (PEA) is a compound that may fill this gap for athletes and active populations. PEA is widely reported to exert analgesic and anti-inflammatory properties [19, 20]. Unlike OTC medications such as ibuprofen and acetaminophen which inhibit the COX pathway, PEA’s pleiotropic effects are likely due to its ability to affect multiple pathways at different receptor sites e.g., proliferator-activated receptor alpha (PPARα) and G protein-coupled receptor 55 (GPR55) receptors and indirectly on cannabinoid receptor type 1 and 2 (CB_1_ and CB_2_); amongst others [21]. Multiple intervention studies have found that PEA doses of between 300 and 1200 mg are effective in the management of pain associated with osteoarthritis and similar dosing relieves headache pain to a similar degree as ibuprofen [22–25]. Additionally, PEA has shown to improve sleep quality and duration, which may be of benefit to athletes as sleep deprivation has shown to negatively impact skeletal muscle hypertrophy and strength performance [26–29]. Some evidence suggests that PEA may also reduce skeletal muscle damage in response to eccentric contractions [23, 25]. Mallard et al. [25] also found that PEA supplementation prior to eccentric exercise led to a significant increase in protein kinase B (PKB) phosphorylation in blood cells. As the activation of Akt/PKB stimulates muscle protein synthesis, these results indicate that if PEA exerted these effects in muscle, it may enhance skeletal muscle mass. Overall, these results suggest that PEA could be an alternative to OTC medications to manage muscular pain in athletes and may even exhibit some additional benefit. To our knowledge, there are no studies that have examined the impact of PEA supplementation on training adaptations during a resistance training program.

This study aimed to explore the effects of daily PEA supplementation (Levagen +) during an 8-week period of resistance training on changes in total body and regional lean body mass (e.g., mid-thigh), strength, power, sleep, wellbeing, and pain associated with resistance exercise training, when compared with a placebo condition. We hypothesized that PEA supplementation together with resistance training does not impair muscle gain and would further improve muscle mass, strength, and power and reduce pain.

## Methods

### Study Design and Participants

In a randomized controlled trial, fifty-two healthy, recreationally active (a minimum of 150 min of self-reported moderate-intensity physical activity per week) participants were recruited. Participants were included in the study if they also were weight stable (for the last 2 months) and had a BMI between 18.5 and 28.0 kg/m^2^. Participants were invited to participate in the study following a screening phone call ensuring that they did not meet the following exclusion criteria:

- Major musculoskeletal injury in the past 6 months
- Participation in regular structured resistance training in the past 6 months (e.g., two or more sessions per week)
- Allergies to any of the contents of the Levagen + PEA supplement or placebo formulation
- Currently participating in a weight loss program or special diet (e.g., low carbohydrate, ketogenic, vegan etc.)
- Current smokers
- Any functional impairment that would limit participation in the intervention
- Current use of sports supplements (e.g., creatine or protein powder) or pain medication known to effect skeletal muscle mass in the last month.

This was a double-blind RCT. The study recruitment and intervention were conducted in 3 cohorts over a 12-month period (from March 2022 to April 2023). The study was approved by the Deakin University Human Ethics Committee (2021–312) and registered with the Australian Clinical Trials Registry (ANZCTR: ACTRN12621001726842p). All participants provided written informed consent before participating in the study. More details regarding the study protocol can be found in our protocol paper [20].

### Measurements

**Fig. 1:**
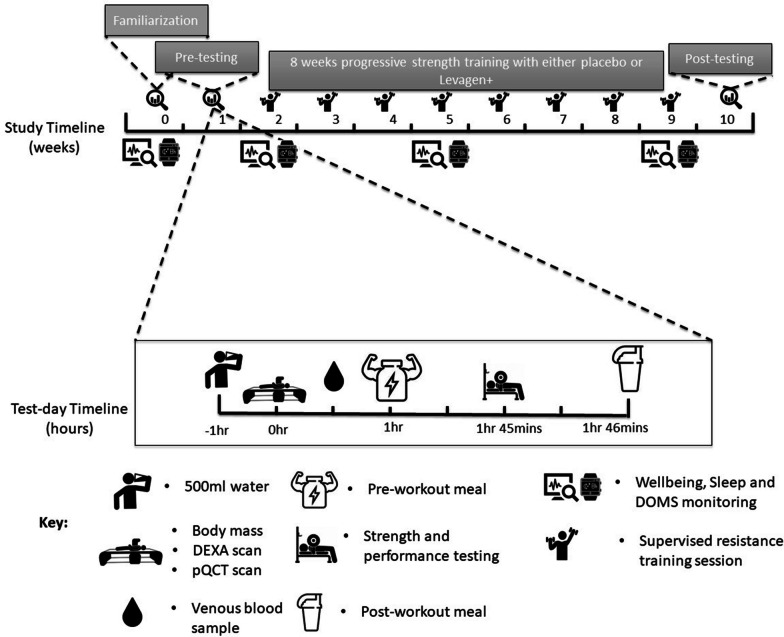
Experimental timeline illustrating the test weeks and the training weeks in addition to the test-day timeline

Participants were asked to attend the laboratory three times, once for familiarization (w0), once at pre-testing (w1) and once at post-testing (w10) (Fig. 1). During the familiarization session consent forms were obtained and basic anthropometric measures were taken. Participant height was assessed using a fixed stadiometer and electronic scales for body mass, these measurements were measured to the nearest 0.1 kg using standardized anthropometrical procedures. The primary outcomes were the mean (± standard deviation, SD) difference between the two groups for total body (%) and regional (e.g., mid-thigh) lean muscle mass. Total body lean mass was assessed using dual-energy X-ray absorptiometry (DXA: DPX-IQ Lunar; Lunar Corporation, Madison, WI). Additionally, relative (%) fat mass (FM), fat-free mass (FFM) and appendicular skeletal muscle mass index (SMI kg/cm^2^) were also assessed as secondary outcomes. Mid-thigh muscle cross-sectional area (mCSA cm^2^) was measured by peripheral quantitative computed tomography (pQCT; XCT 3000, Stratec Medizintechnik GmbH, Pforzheim, Germany). A tomographic slice was taken at 66% of femur length proximal to the tibial condyle on the right leg. The pQCT results (muscle cross-sectional area cm^2^ and muscle density cm^3^) were analysed using BoneJ plugin for ImageJ. The coefficient of variation value (CV) was determined for DXA (Mean ± SD% PEA: 0.65 ± 0.49; PLA: 0.70 ± 0.55) and pQCT (Mean ± SD% PEA: 2.1 ± 2.3% PLA: 1.7 ± 1.8%) using the values for total and regional lean mass measured over two consecutive weeks (w0 and w1).

### Intervention

Participants were provided with capsules containing 175 mg PEA Levagen + (containing not less than 150 mg of PEA) or identically matched placebo (PLA) pills containing maltodextrin, which were to be taken twice a day for 8 weeks. Participants were provided these along with a standardized pre-workout snack and instructed to take 1 × capsule 60 min prior to exercise with their pre-workout snack (muesli bar and breakfast drink, provided a total of: 388 kcals, 45 g carbohydrates, 21 g protein, 13 g fat) on training days or with their breakfast on non-training days, and another capsule in the evening 60 min prior to sleep each day. PEA and PLA supplements were identical in appearance, marked as “Group A” or “Group B” and were provided by Gencor Pacific Ltd with the blind retained by an independent staff member not associated with the study. Participants were asked to track their pill ingestion using a provided handwritten tracking sheet and to return unemptied pill bottles which were counted to confirm compliance.

Allocation of the groups was carried out by a biostatistician that was blinded to the contents of the supplements and generated group allocation via stratified block randomization, with block sizes of two and stratification factors being biological sex and appendicular SMI [SMI kg/cm^2^ male, low SMI (≤ 8.09 kg/cm^2^); male, high SMI: (> 8.09 kg/cm^2^); female, low SMI (≤ 6.64 kg/cm^2^); female high SMI (> 6.64 kg/cm^2^]. These values were determined based on the average SMI measured within the cohort. SMI was used as a stratification to ensure there was even groups for baseline strength, as SMI can be a predictor of muscle strength [30]. The randomization allocations were produced with Stata module *ralloc* and participants were allocated to their groups by a researcher not associated with the study. The blind was not broken until after the data analysis was completed.

Participants were provided with a 500 ml bottle of water to consume 45–60 min prior to DXA to control hydration status and to minimize the influence of hydration status on DXA-derived measures. Prior to the testing week (w1) participants were asked to fill in a 24-h food diary for the day prior to testing and asked to ingest the same food and fluids again the day before the post-test day of the study to attempt to control for dietary variables. Participants were required to attend the laboratory between 7.00 am and 9.00 am in an overnight fasted, euhydrated state as confirmed by blood osmolarity testing (w1: 281 ± 11.4 mOsmol/kg; w10: 281 ± 9.7 mOsmol/kg (*p* = 0.74); Gonotec Osmomat 3000 Basic, Berlin, Germany) and avoiding any strenuous exercise in the 24-h prior to testing. Heparin whole blood samples were taken and these samples were centrifuged at 4000 rpm for 10 min within 15 min of sample collection. 2 × μL of plasma was used to determine plasma osmolality in duplicate (CV = 0.9%).

Prior to any exercise testing, participants were provided a standardized breakfast (muesli bar and breakfast drink, provided a total of: 388 kcals, 45 g carbohydrates, 21 g protein, 13 g fat).

### Countermovement Jump and Isometric Mid-thigh Pull

A countermovement jump (CMJ) and isometric mid-thigh pull (IMTP) were carried out using a force plate (400 s + Performance Force Plate, Fitness Technology, Adelaide, Australia) and analyzed using Ballistic Measurements System software (BMS: Fitness Technology, Skye SA). Following a standardized warm-up protocol (3 × 3 estimated submaximal effort CMJ; 50%, 70% and 90%), participants performed three maximal-effort CMJs on the force plate, with 2-min rest between sets. To perform the CMJ participants were asked to self-select their squat depth before accelerating as quickly as possible from the bottom to achieve maximal jump height. The jump with the highest peak power (W) was used. Other variables such as peak force (N) and jump height based on flight time were also reported from the highest peak power variable.

IMTP was performed on the force plate using an adjustable bar and using standard methodological guidelines as previously outlined [31]. Bar positions were standardized for each participant using hip (140–150°) and knee angles (125–145°), which were measured using a goniometer determined during the familiarization session. Participants were secured using lifting straps, using a clean grip, upright torso, slight flexion in the knee and with feet centered under the bar approximately hip width apart. Minimal pre-tension was allowed to ensure that there was no slack in the participants’ body and the bar prior to the initiation of the pull. Prior to maximal testing, a standardized warmup protocol (3 × 3 estimated submaximal effort IMTP; 50%, 70% and 90%) was performed. For the maximal attempt, participants were asked to pull “as hard and as fast as possible” for a 3-s maximal voluntary isometric contraction after a 3-s countdown. Participants were allowed 2-min rest between sets and performed three maximal efforts of IMTP. The trial with the greatest peak force (N) was selected for statistical analysis.

### Bench Press Throw

The bench press throw was performed using a Smith Machine (Maxrack, IP-L8505, Star trac, Irvine, California, United States), with the empty barbell weighing 18 kg. Kinematic data was measured using a linear position transducer (GymAware v2.4.1, GymAware, Kinetic Performance Technology, Canberra, Australia) which was attached to the end of the barbell knurling. Participants first followed a standardized warm-up (4 reps slow and controlled, 4 reps at 50% max effort; 4 reps at 75% max effort; 4 reps at 90% max effort). Participants were then asked to perform four maximal-effort bench press throws, accelerating as quickly as possible out from their chest. Participants rested for 2-min between sets and performed this three times. The highest value for maximum power (W) was recorded for each participant.

### 1-Repetition Maximum Bench Press

Participants then performed a 1-repetition maximum (1-RM) using a fixed bench press. Following a standardized warm-up (8, 5, and 3 repetitions at 50%, 75%, and 95% of predicted 1-RM respectively), participants performed 1-RM attempts with increasing loads (5–10 kg for males, 2.5–5 kg for females), with a 2.5 kg minimum load, until the 1-RM was reached. Participants rested for 3–5 min between sets and were allowed to have 2 re-attempts at a failed lift. Participants’ hands were in a closed pronated grip and positioned slightly wider than shoulder-width apart to allow for a 90-degree elbow angle at the bottom of the range. The participants were assisted to get the bar from the support racks to the start position with the arms fully extended above the chest. The participants then lowered the bar slowly to their chest or slightly above (e.g., a fist length) their chest and then returned the bar to a fully extended position (e.g., full elbow extension) for it to be considered a valid lift.

### Questionnaires

#### Perception of Pain, Wellbeing and Sleep Quality

Questionnaires were provided to participants using a QR code to complete via an online database and survey platform (REDcap) on their own personal phones. Questionnaires were completed at baseline (week 0), week 2, week 5 and week 8 prior to any exercise session. Questions regarding pain included a 10-point (0–10) visual analogue scale (VAS) where participants were asked to monitor pain associated with delayed onset muscle soreness (DOMS) (0 = no pain; 1–3 = mild pain; 4–6 = moderate pain; 7–10 = severe pain) [32].

The short recovery and stress scale measures the recovery-stress state of an athlete [33]. In the current study it was used as a proxy for wellbeing to track athlete responses to the training. This eight-item questionnaire offers differentiated information regarding the recovery-stress state of a person, with response options on a 6-point scale (e.g., 0 = does not apply at all to 6 = fully applies). For the four items related to recovery, participants were asked to rank their current state of recovery (i.e., physical performance capability mental performance capability; emotional balance; overall recovery). For the items related to perceptions of stress (i.e., muscular stress, lack of activation, negative emotional state, overall stress) participants were asked to rank their current state of stress. The higher the value for ranked item, the higher the perceived recovery or stress state.

The Pittsburgh sleep quality index (PSQI; Buysse et al. [34]) is a 19-item self-rated questionnaire for evaluating subjective sleep quality. The 19 questions are combined into 7 clinically derived component scores, each weighted equally from 0 to 3. The 7 component scores are added to provide a global score (0–21). PSQI scores that are > 5 indicate poor sleep quality. Sleep onset latency (SOL) was also determined using the PSQI and reported within the results to determine if there were any changes between groups across the study.

#### Physical Activity

Physical activity was measured using an ActiGraph (ActiGraph GT9X, ActiGraph, Pensacola, FL) which participants wore on their wrist on their non-dominant side. The participants were instructed to wear the activity watches for the whole study trial period and to remove the watch only for engaging in aquatic activities, engaging in contact sports or when charging was required. Data was processed using ActiLife software (v6.13.4; ActiGraph Corp., LLC). Physical activity measured by the accelerometer was examined in four ways: (1) minutes per day (min/d) spent MVPA; (2) physical activity energy expenditure (PAEE, kcal/kg/day); (3) steps per day (steps/day); (4) total activity counts per day (TAC/d). Data from baseline (week 0), week 2, week 5, and week 8 were collected to align with the questionnaires. A valid day was determined by wear time of > 10 h/day or 80% wear time, 3-days were considered a minimum number of valid days for the week or collected data [35, 36].

#### Food Diaries

Participants were asked to maintain their habitual food intake during the course of the study period. Two-day food and fluid diaries (non-consecutive days: 1 weekday and 1 weekend) were filled out twice during the study (week 2 and week 8). Diaries were analyzed using FoodWorks v.10 nutritional analysis software (Xyris Software, 2019, Brisbane, Australia) based on the Australian Food Composition Database (AUSNUT 2019). The average total daily energy and macronutrients (e.g., protein, carbohydrates, and fats) were reported from the two days.

#### Resistance Training Protocol

Participants completed 8-weeks of supervised progressive resistance training, that took place on two non-consecutive days at the University campus. A standardized snack (388 kcals, 45 g carbohydrates, 21 g protein, 13 g fat) was provided for participants to consume 45–60 min prior to attending their training session. The exercise sessions lasted 65 ± 12 min and were supervised by a qualified exercise coach (e.g., exercise physiologist or qualified strength and conditioning coach) to monitor correct lifting and rest intervals. There were 8 exercises that were completed as four complex pairs (The exercises and complex pairs include: 1A leg press + 1B countermovement jump, 2A bench press + 2B bench press throw, 3A deadlift + 3B deadlift jumps, and 4A bench pull + 4B power bench pull. The strength-focused exercises (denoted with letter “A” of each complex pair) were performed with a slow and controlled eccentric phase of ~ 2–3 s for increasing time under tension (stimulus for hypertrophy) and an explosive concentric phase (stimulus for strength and power development) with higher loads. Loads for weeks 1–2 were set at 75% and 45% of 1-RM (or predicted 1-RM) for the strength and power-focused exercises, respectively, with sets and reps at 3 × 6. The loads for the exercises increased by 5% every two weeks and the sets and reps were adjusted as follows: 3 × 5, 4 × 4 and 5 × 3. During weeks 3–8, an additional set was performed after each of the strength-focused exercises, whereby the load was decreased (to 50–60%, 1-RM) and participants then performed as many reps as possible (AMRAP). Additional accessory exercises (e.g., leg curls, bicep curls and triceps pushdowns) were performed at the end of every session (3 × 8–10, sets and reps) at a 7–8 rating of perceived exertion (RPE), on a 10-point scale to allow for muscle balance. Rest between sets and exercises were 3–5 min. More details regarding the exercise protocol can be found in Huschtscha et al. [20]. Participants were asked to rate their perceived exertion (RPE) on a 10-point scale for each session, and the session duration was also recorded to calculate the session-RPE. After the completion of the resistance training session, participants were provided with a protein recovery beverage (250 kcals; 40 g protein) that was consumed within 15 min of completing the session.

#### Statistical Analysis

To achieve statistical significance, a sample size of 42 participants (21 per group) was determined to provide 80% power, α = 0.05, to detect a 2% difference between groups in percentage lean mass change (assumed SD = 2.2% based on data from [37]]. Sample size calculations were performed in Stata/SE 16.1 (StataCorp, TX) using the *power two means* command.

Descriptive statistics were calculated for all variables, with mean ± standard deviation reported for continuous variables and counts and percentages reported for categorical variables. Within-group changes over time were analyzed using paired-sample t-tests. No adjustment for multiplicity was undertaken in the analysis of primary or secondary outcomes. Instead, 95% confidence intervals and *p*-values have been reported alongside representing the data with individual responses illustrated to enable readers to use their own judgment about the relative weight of the conclusions on the effect of PEA on the various outcomes. This approach aligns with the usage of *p*-values favoured by the American Statistical Association [38].

Analysis of intervention effects were conducted on both an intention-to-treat (ITT) (with all participants included in analyses according to their allocated group) and a per-protocol (PP) basis which included participants who attended ≥ 80% of their resistance training sessions and consumed ≥ 80% of their supplements (PEA and PLA). As there was no/minimal missing data for the main outcomes, intervention effects (i.e., between group differences at post-testing) for these outcomes were estimated using linear regression models with group as the independent variable and sex, SMI group (high/low), and baseline levels of the outcome as covariates. Differences between groups for outcomes that had only one measurement point (e.g., % compliance) were estimated using linear regression models with group as the independent variable and sex and SMI group (high/low) as covariates. Other outcomes (diet, PA, pain, stress, sleep, plasma osmolality) were analyzed in long-format using linear mixed models that allowed all participants who provided at least one follow-up observation to be analyzed. These mixed models used baseline measurements as part of the outcome with an unstructured variance-covariate matrix (allowing for baseline level to be adjusted for) and included terms to estimate intervention effects at each follow-up point while adjusting for sex and SMI group (high/low). Normality was assessed via inspection of histograms of residuals from between-group analysis models, and this assumption appeared reasonable for all outcome models.

## Results of Participants

**Fig. 2:**
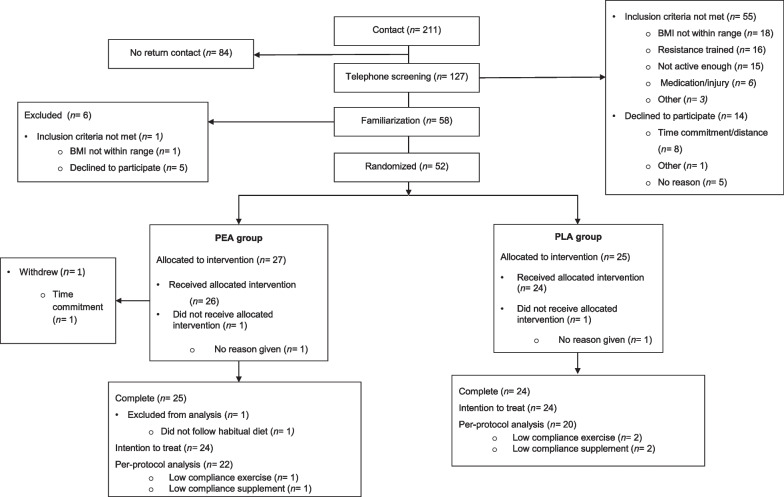
CONSORT (A Consolidated Standards of Reporting Trials) Flow diagram. Flow-chart showing inclusion, randomization and participation throughout the study

A total of 127 potential participants were screened via the telephone; 58 were deemed eligible and underwent a familiarization screening, five of whom declined to participate and n = 1 had a higher BMI than reported over the phone and 52 were randomized. A total of n = 49 participants completed the 11-week study. One participant from the PEA group was excluded from the study analysis after reporting he was intentionally in a caloric deficit for the duration of the study, which violated study protocol instructions to maintain habitual diet. Therefore, 48 participants were included in the final analysis based on intention to treat (ITT) (n = 24, PEA; n = 24 PLA) and 42 (n = 22, PEA; n = 20 PLA) were included in the per-protocol analysis based on > 80% compliance for exercise and supplementation. A Consolidated Standards of Reporting Trials (CONSORT) diagram is shown in Fig. 2.

The baseline characteristics for the two groups are shown in Table 1.

**Table 1:** Baseline participant characteristics

	PEA (n = 24)	PLA (n = 24)	*p*-value
	Mean ± SD	Mean ± SD	
Sex, n (%)			0.56
Male	9 (38%)	11 (46%)	
Female	15 (63%)	13 (54%)	
Age (yrs)	22 ± 4	23 ± 5	0.42
SMI (kg/m^2^)	7.00 ± 0.97	7.18 ± 0.79	0.48
Height (m)	1.72 ± 0.12	1.72 ± 0.09	0.99
Weight (kg)	68.02 ± 14.05	68.00 ± 11.36	1.00
BMI (kg/m^2^)	22.80 ± 2.49	22.87 ± 2.34	0.92
DXA, body fat (%)	27.31 ± 8.18	24.89 ± 6.50	0.26

*PLA* placebo, *PEA* palmitoylethanolamide, *SMI* skeletal muscle mass index, *BMI* body mass index, *DXA* dual-energy X-ray absorptiometry

### Adherence and Session-RPE

The average exercise training compliance based on the values for ITT was 92.0 ± 7.7% and 92.1 ± 10.1% for the PEA and PLA groups respectively, with no statistically significant differences between groups (ITT: *p* = 0.670; PP: *p* = 0.450). Average adherence to the supplements was 93.0 ± 12.8% and 94.5 ± 7.7% for the PEA and PLA groups respectively (ITT: *p* = 0.460; PP: *p* = 0.260). There were no adverse effects reported. Thirty seven percent (n = 9) of participants correctly identified that they were in the in-supplement group, whereas 58% of participants correctly identified that they were taking the placebo. Reported session training load [RPE x session duration (min)] for PEA (496 ± 92 AU) and PLA (467 ± 91 AU) was not significantly different between groups (ITT: *p* = 0.250; PP: *p* = 0.590).

### Anthropometric and Lean Muscle Mass

**Fig. 3:**
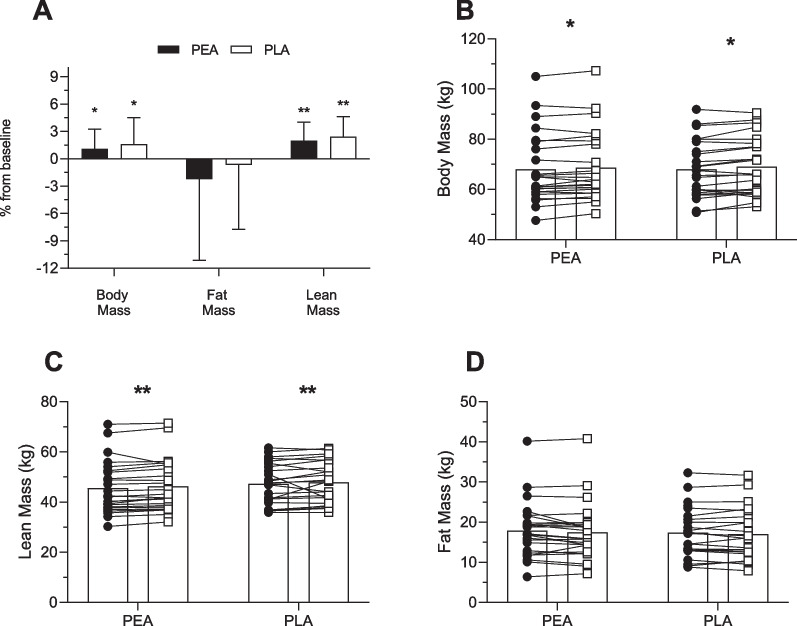
Group (**A**) and individualized changes in total body mass (**B**)**;** lean body mass (**C**)**;** and fat mass (**D**) from baseline in PEA and PLA supplement groups. Pre (●) and post (○). Values are means ± SD (PEA, *n* = 24*;* PLA*, n* = 24) **p < 0.01 and *p < 0.05 vs. baseline

**Fig. 4:**
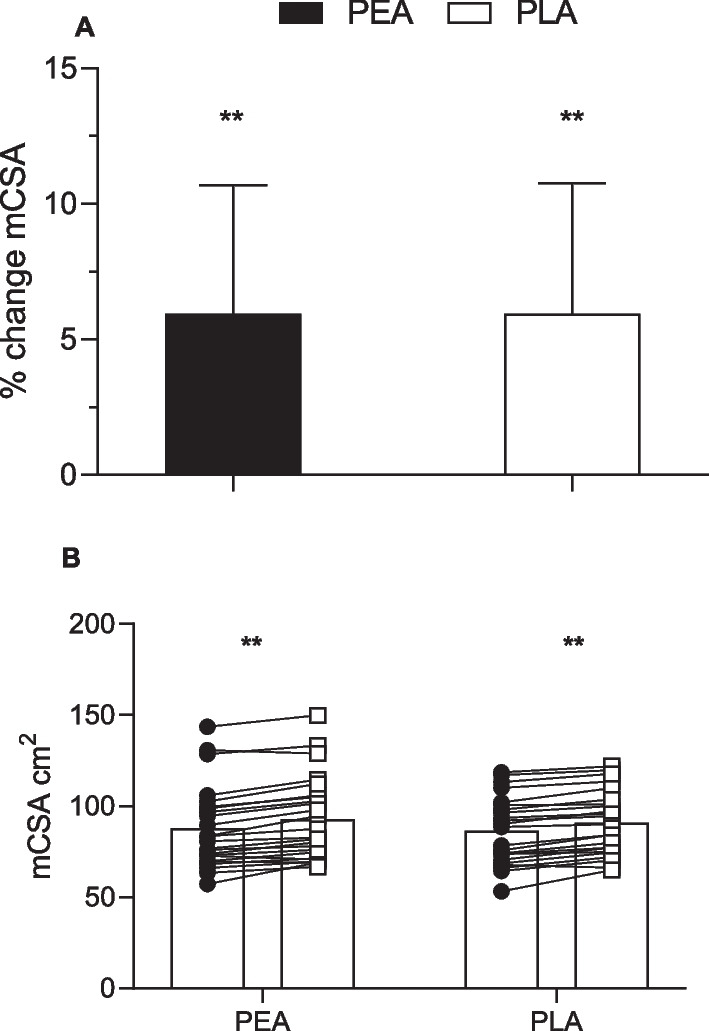
Group (**A**) and individualized (**B**) changes in muscle cross sectional area (mCSA) of the mid-thigh from baseline PEA and PLA supplement groups. Pre (●) and post (○). Values are means ± SD (PEA, *n* = 24*;* PLA*, n* = 23) **p < 0.01 vs. baseline

Body mass increased in both groups over the 8 weeks (PEA: 0.6 ± 1.4 kg; PLA: 1.0 ± 1.8 kg), with no significant difference between groups (Table 2; Fig. 3B). Lean mass increased by 2.0 ± 2.0% and 2.4 ± 2.1% in the PEA and PLA groups respectively, with no significant differences between groups (ITT: *p* = 0.38; PP: *p* = 0.39; Table 2; Fig. 3C). Mid-thigh mCSA increased by 5.9 ± 4.7% and 5.9 ± 4.8% in the PEA and PLA groups respectively but there were no significant between group differences (ITT: *p* = 0.58; PP: *p* = 0.53 Fig. 4A and B). There was no significant between group difference for change in FM (kg) (PEA: − 2.24 ± 8.90; PLA: − 0.66 ± 7.09) (Fig. 3D and Table 2).

**Table 2:** Pre- and post-testing values and the mean within-group changes for anthropometry, total and regional lean mass, strength and physical performance outcomes according to randomized allocation

	PEA	PLA	Group difference (ITT)^a^	Group difference (PP)^b^
	Mean ± SD	Mean ± SD	B [95% CI) *p*-value	B [95% CI) *p*-value
Anthropometry and total and regional lean mass outcomes				
Weight (kg)				
Pre-testing (W1)	68.02 ± 14.05	68.00 ± 11.36	0.33 (− 0.61, 1.27) 0.48	0.18 (− 0.86, 1.22) 0.73
Post-testing (W10)	68.67 ± 13.91	69.05 ± 11.39		
% change	1.09 ± 2.15	1.62 ± 2.88		
Within group change	0.66 ± 1.40	1.05 ± 1.85		
Lean muscle mass (kg)				
Pre-testing (W1)	45.47 ± 10.28	46.79 ± 8.26	0.25 (− 0.31, 0.81) 0.38	0.25 (− 0.34, 0.84) 0.39
Post-testing (W10)	46.33 ± 10.31	47.89 ± 8.24		
% change	2.00 ± 2.03	2.43 ± 2.19		
Within group changes	0.86 ± 0.88	1.10 ± 1.02		
Fat mass (kg)				
Pre-testing (W1)	17.95 ± 7.07	17.40 ± 6.21	0.36 (− 0.39, 1.11) 0.33	0.24 (− 0.55, 1.03) 0.54
Post-testing (W10)	17.47 ± 7.12	17.29 ± 6.29		
% change	− 2.24 ± 8.90	− 0.66 ± 7.09		
Within group changes	− 0.48 ± 1.41	− 0.11 ± 1.06		
mCSA (cm^2^)				
Pre-testing (W1)	87.72 ± 22.45	86.22 ± 18.37	− 0.56 (− 2.57, 1.45) 0.58	− 0.70 (− 2.97, 1.57) 0.53
Post-testing (W10)	92.73 ± 22.95	90.91 ± 17.80		
% change	5.96 ± 4.73	5.94 ± 4.83		
Within group changes	5.01 ± 3.60	4.69 ± 2.89		
mDCSA (mg/cm^3^)				
Pre-testing (W1)	79.43 ± 2.30	79.57 ± 2.40	− 0.59 (− 1.39, 0.21) 0.14	− 0.45 (− 1.27, 0.37) 0.28
Post-testing (W10)	79.71 ± 1.68	79.23 ± 1.41		
% change	0.38 ± 1.90	− 0.36 ± 3.15		
Within group changes	0.28 ± 1.53	− 0.34 ± 2.59		
Strength and performance outcomes				
CMJ: Jump height (cm)				
Pre-testing (W1)	25.16 ± 6.82	26.12 ± 5.77	− 2.94 (− 5.15, − 0.74) 0.010	− 2.93 (− 5.31, − 0.55) 0.017
Post-testing (W10)	29.38 ± 8.59	27.43 ± 6.26		
% change	17.33 ± 17.46	5.27 ± 10.39		
Within group changes	4.22 ± 4.37	1.31 ± 2.80		
CMJ: Peak force (N)				
Pre-testing (W1)	1445.42 ± 314.74	1545.06 ± 383.12	31.81 (− 95.34, 158.96) 0.62	15.24 (− 125.42, 155.91) 0.83
Post-testing (W10)	1467.49 ± 373.79	1575.88 ± 372.99		
% change	2.49 ± 20.38	2.44 ± 9.98		
Within group changes	22.07 ± 307.74	30.83 ± 163.37		
CMJ: Peak power (W)				
Pre-testing (W1)	3042.00 ± 1094.08	2932.06 ± 1022.03	− 315.84 (− 721.80, 90.12) 0.12	− 378.79 (− 827.25, 69.67) 0.095
Post-testing (W10)	3453.03 ± 1185.80	3147.34 ± 923.10		
% change	17.95 ± 30.55	10.33 ± 20.79		
Within group changes	411.02 ± 964.13	215.28 ± 522.40		
IMTP: Peak force (N)				
Pre-testing (W1)	1787.99 ± 623.87	1865.87 ± 524.93	− 40.12 (− 170.31, 90.07) 0.54	− 63.42 (− 192.62, 65.78) 0.33
Post-testing (W10)	1993.65 ± 696.93	2037.17 ± 553.35		
% change	11.74 ± 11.63	10.39 ± 14.79		
Within group changes	205.66 ± 194.89	171.30 ± 246.37		
Bench Throw: Peak power (w)				
Pre-testing (W1)	439.96 ± 193.96	496.83 ± 207.91	− 8.20 (− 42.38, 25.98) 0.63	− 6.32 (− 42.90, 30.26) 0.73
Post-testing (W10)	506.29 ± 211.22	551.04 ± 207.73		
% change	16.12 ± 11.43	13.59 ± 14.22		
Within group changes	66.33 ± 56.96	54.21 ± 59.11		
1-RM bench press (kg)				
Pre-testing (W1)	42.60 ± 16.28	44.58 ± 15.87	2.24 (0.12, 4.37) 0.039	2.73 (0.40, 5.06) 0.023
Post-testing (W10)	49.17 ± 15.35	53.33 ± 16.82		
% change	18.67 ± 12.63	21.33 ± 9.03		
Within group changes	6.56 ± 3.89	8.75 ± 3.13		

*1-RM* 1 repetition maximum, *PLA* placebo, *CMJ* countermovement jump, *IMTP* isometric thigh-pull, mCSA, muscle cross-sectional area; mDSA, muscle density sectional area; PEA, palmitoylethanolamide

^a^Mean difference between groups at final testing week, estimated using linear regression models adjusting for baseline outcome level and stratification factors sex and SMI (low/high), among all participants that completed the trial (intention to treat analysis; n = 48 for all outcomes except for muscle cross sectional area which had n = 47)

^b^Mean difference between groups at final testing week, estimated using linear regression models adjusting for baseline outcome level and stratification factors sex and SMI (low/high), among participants that attended ≥ 80% of their resistance training and consumed ≥ 80% of their supplement over the course of the study (per-protocol analysis; n = 42 for all outcomes except for muscle cross sectional area which had n = 41

### Lower Body Strength and Power

**Fig. 5:**
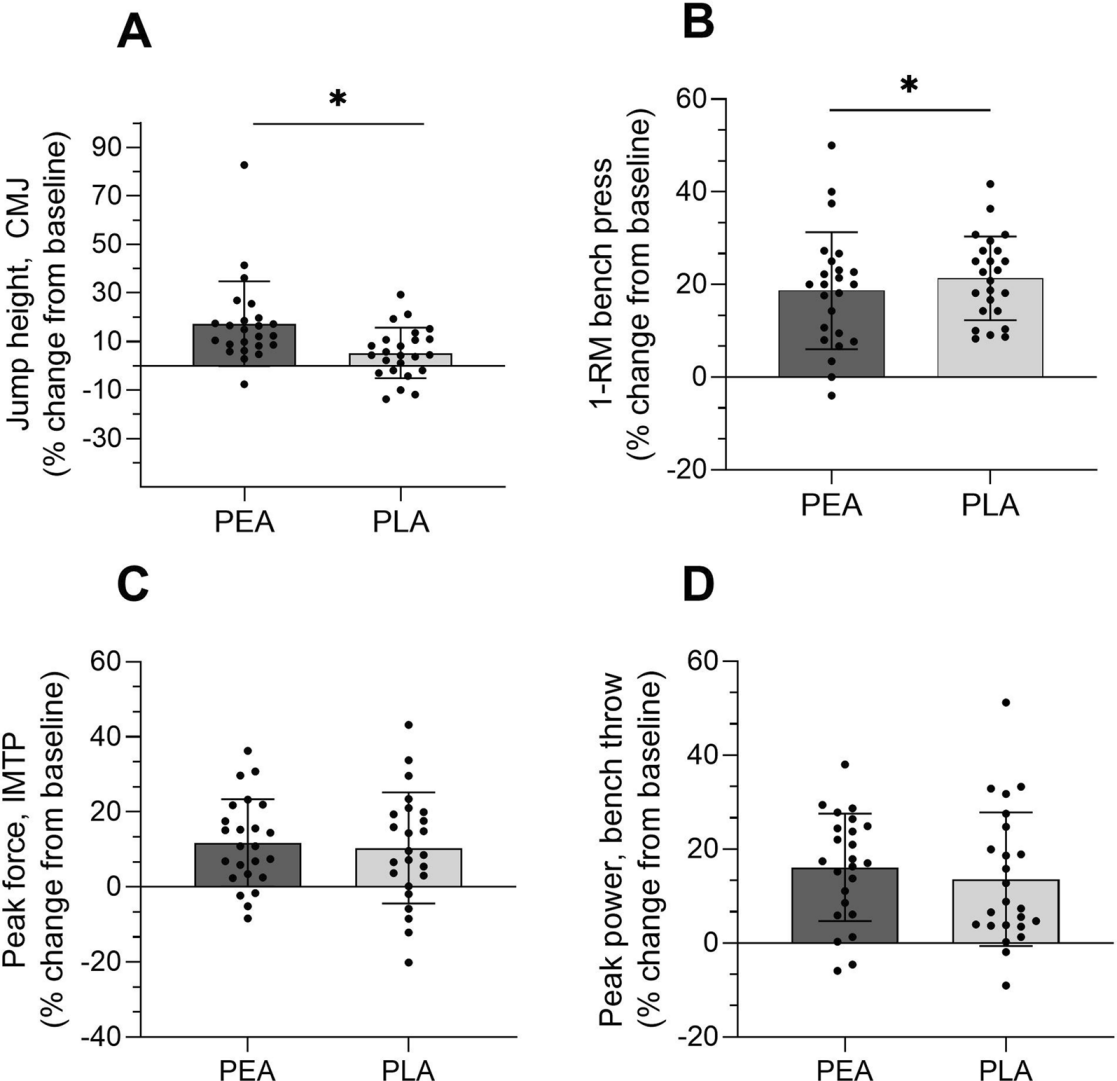
Pre to post percentage change (**A**) Jump height (**B**) 1RM bench press; (**C**) Peak force for isometric mid-thigh pull; (**D**) Peak power bench throw. Values are presented as mean percentage change ± SD (with individual data points), Asterisk denotes significant differences between the two groups (*p* < 0.05). PEA and PLA supplement groups. (PEA, *n* = 24*;* PLA*, n* = 24)

Both the PEA group (17.3 ± 7.4%) and PLA group (5.2 ± 10.3%) had increased mean jump height from baseline, with significantly higher jump height (CMJ) at week 10 in the PEA group compared to the PLA (Adjusted mean difference [95% CI] *p*-value; ITT: − 2.94[[− 5.15, − 0.74] *p* = 0.010; PP: − 2.93 [− 5.31, − 0.55] *p* = 0.017), respectively (Table 2; Fig. 5A). There were no significant differences between groups for IMTP outcomes (Table 2; Fig. 5B).

### Upper Body Strength and Power

Both groups increased 1-RM bench press from baseline (PEA = 18.67 ± 12.63%; PLA = 21.33 ± 9.03) (Table 2; Fig. 5C). The PLA group had higher 1-RM bench press post-intervention compared with the PEA group (ITT: 2.24 [0.12, 4.37] *p* = 0.039); PP: 2.73 [0.40, 5.06] *p* = 0.023). There were no significant differences between groups for outcomes relating to upper body power (Table 2; Fig. 5D).

### Subjective Pain, Recovery, and Stress Scores

**Fig. 6:**
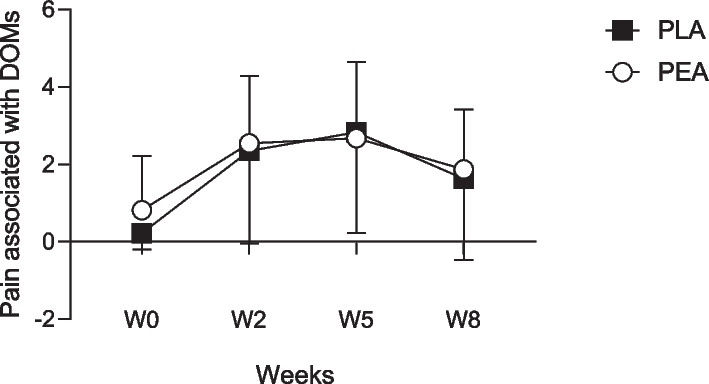
Subjective pain associated with muscle soreness, weeks 0,2,5 and 8. Values are presented as group mean ± SD. ○ PEA, *n* = 24*; ■* PLA*, n* = 24

**Fig. 7:**
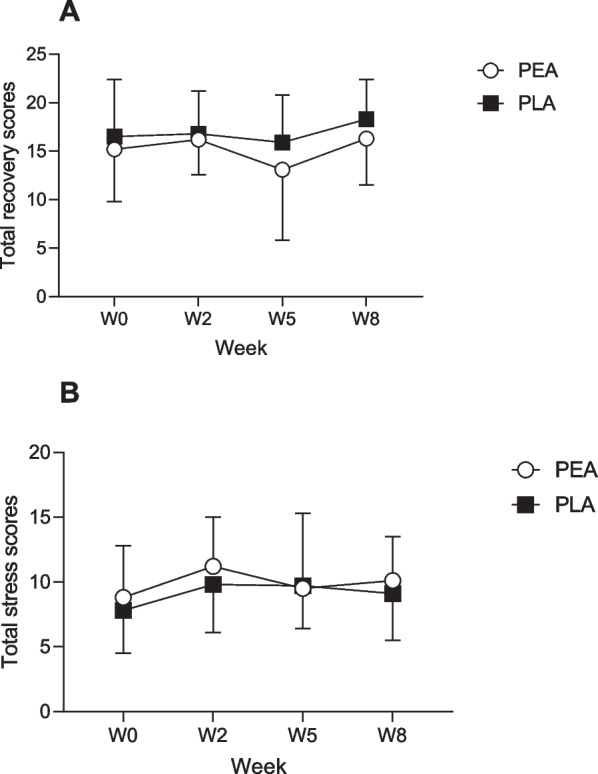
Subjective scores relating to (**A**) Total recovery scores (**B**) Total stress scores for ○ PEA, (*n* = 24) and *■* PLA (*n* = 24) at baseline, weeks 2,5,8**.** Values are presented as group mean ± SD

Reported subjective pain associated with DOMS at baseline (week 0) was 0.82 ± 1.40 and 0.22 ± 0.42 for PEA and PLA groups respectively. There were no significant differences between the groups at any of the time points (Fig. 6). There were no significant between group differences in total recovery scores, which are an accumulative score of physical capacity, muscle performance, emotional balance and overall recovery (Fig. 7A). Similarly, there were no significant between group differences in the short stress scale which includes muscular stress, lack of activation, negative emotional state and overall stress (Fig. 7B).

### Sleep

**Fig. 8:**
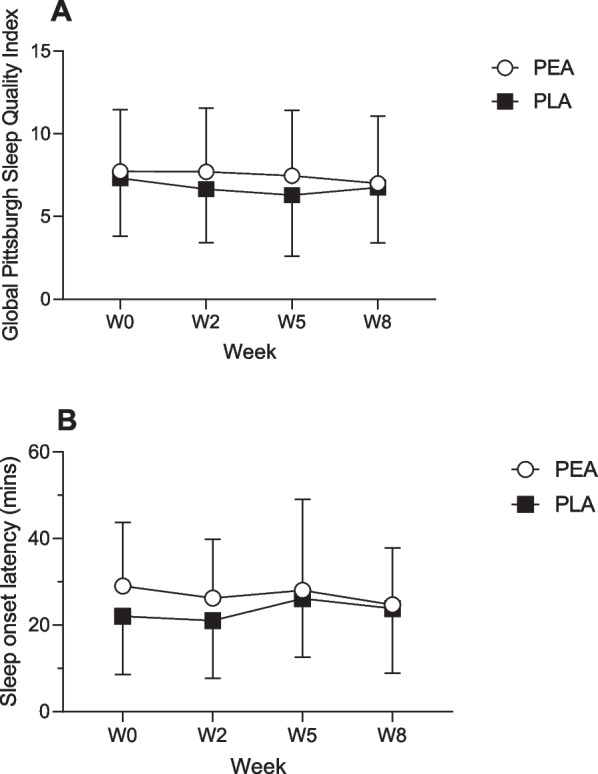
Subjective sleep quality based on (**A**) Pittsburgh sleep quality index and (**B**) Self-reported sleep onset latency for the ○ PEA, (*n* = 24) and *■* PLA (*n* = 24) group at baseline, 2, 5 and 8 weeks

The Pittsburgh sleep quality index (PSQI) scores over the study period for both groups are presented in Fig. 8A. At baseline, PSQI scores > 5 were 78% and 89% for the PEA and PLA groups respectively indicating “poor” sleep. There were no significant between group differences PSQI scores across the study. Self-reported sleep onset latency (SOL) at baseline was 29 ± 15 min and 22 ± 13 min in the PEA and PLA group respectively (Fig. 8B). However, there were no significant between group differences in SOL.

### Food Diaries and Physical Activity

Energy intake for both groups did not change significantly when recorded at baseline and 8 weeks for either group (PEA: 1988 ± 764 kcals, *p* = 0.934; PLA: 1910 ± 379 kcals, *p* = 0.918). Similarly, reported protein intakes were consistent for both groups at baseline and 8-weeks (1.40–1.55 g/kg BM/day) with no differences between groups (Supplementary Table 1).

Outcomes for physical activity are presented in Supplementary Table 2. There were no significant changes across the study time or between groups at any of the time points for average steps, kcals/day, minutes in moderate to vigorous physical activity, or total activity counts.

## Discussion

To our knowledge, this is the first study to investigate the effects of a daily Levagen + PEA supplementation on the adaptive responses to strength training. Our findings indicate that 2 × 175 mg daily of Levagen + combined with an 8-week progressive resistance training program resulted in a significant improvement in dynamic lower body power as assessed by CMJ. However, contrary to our hypothesis, PEA supplementation did not enhance muscle hypertrophy, upper body strength, upper body power or lower body isometric strength. The practical implications of these novel findings indicate that, unlike NSAIDs and other analgesics that have been shown to inhibit strength training adaptations, moderate doses of Levagen + PEA do not negatively affect hypertrophy or power adaptations and may improve lower body power in recreationally active individuals.

Strength training combined with the standardized peri-workout nutrition was successful at increasing total lean mass (0.8–1.1 kg). While the fat mass reduction was not significant across time (− 0.6 to –2.2%), the incongruous increase in total body mass and total lean mass suggests that our participants achieved a degree of favourable body re-composition with the majority of the weight gain being in the form of lean mass. In addition to significant changes in total lean mass, we also detected significant changes in regional mCSA (4.7–5.0 cm^2^) as assessed by pQCT. These anthropometric findings are aligned with a systematic review from Morton et al. [39] demonstrating that resistance training alone (> 6 weeks) results in an increase in lean mass (1.1 ± 1.2 kg) and an increase in mid-femur (5.2 ± 3.0 cm^2^) mCSA. Therefore, the training program and nutritional support was sufficient to induce improvements in lean mass and muscle hypertrophy similar to previous studies of a similar design. The increase in whole body mass is consistent with the pre-workout and post-workout nutrition we provided to maximize muscle mass gains which provided an additional 776 kcals per week where similar amounts of weight gain have previously been demonstrated in overfeeding studies with resistance training supporting that the program was successful in inducing muscle hypertrophy [40]. However, there were no significant between group differences in any of the anthropometric outcomes. Whilst these findings are contrary to our hypothesis, they do support the notion that unlike ibuprofen, PEA does not exert an inhibitory influence on muscle hypertrophy.

For outcomes relating to lower body power and isometric strength, we observed that the Levagen + PEA group showed a significant improvement in countermovement jump height compared to the placebo group (17% vs 5%; PEA and PLA group respectively), indicating that PEA may augment dynamic lower body power. Previous studies that have determined the reliability of CMJ, found jump height to have between-day reliability of < 5% based on coefficient of variation [41]. Therefore, the change between groups is greater than the error of the measurement. We should note that we have not adjusted for multiplicity in our analysis. That said, a potential explanation as to why there was a significant increase in dynamic lower body power in the absence of differences in muscle hypertrophy could relate to PEA supplementation improving neuromuscular function.

Indirect evidence of a neuromuscular effect of PEA comes from integrative work on muscle from patients with Amyotrophic Lateral Sclerosis (ALS) [42, 43]. This work demonstrated that PEA may target the acetylcholine (ACh) receptor by preventing the desensitization of acetylcholine-evoked currents. This protective effect of PEA on ACh receptor run down was associated with reduced respiratory fatigue in ALS patients treated with PEA [43]. Furthermore, in patients with myasthenia gravis, 1200 mg of oral supplementation with PEA improved the patients’ response to repetitive nerve stimulation on the masseteric nerves resulting in improved disease severity scores and a decrease in muscular fatigue [44]. These data indicate that PEA may also regulate neuromuscular function in certain contexts. Although highly speculative, the significant improvement in dynamic lower body power in the PEA group could be due to the interaction of PEA with muscle ACh receptors, leading to an increase in neuromuscular function. More studies in healthy active individuals should explore this potential mechanism as an application for PEA as an ergogenic aid.

For upper body strength and power, we found that the PEA group had a small but significantly lower improvement in bench press 1-RM than the PLA group. However, the magnitude of the effect (2.2 kg or 2.7%) between groups is smaller than the minimum increments between bench press attempts (2.5 kgs) and is within the coefficient of variation for 1-RM testing (e.g., 4.1%) [45]. Overall, while there were statistically significant differences in 1-RM bench press favouring the PLA group, considering the magnitude of the effect and technical error it is likely that the bench press effect is not meaningful. This is further supported by the findings that the increase in bench press peak power was not different between groups.

We observed no significant difference in self-reported pain or wellbeing outcomes between the PEA and PLA groups over the course of the study. Similarly, Mallard et al. [25] observed no significant difference in exercise associated pain in a cohort of 20 young (untrained) males undergoing strength training in an acute setting. However, a recent systematic review and meta-analysis that included 11 studies and 774 participants, found that PEA supplementation (400–1200 mg PEA) was associated with a 1.68-fold reduction in self-reported pain compared to control [22]. This review included studies of participants who had chronic medical and pain conditions (e.g., multiple sclerosis, osteoarthritis), therefore the mechanisms of the pain and pain tolerance of the subjects in the meta-analysis may be different to the mechanisms of pain in exercise studies. Furthermore, the use of recreationally active individuals in combination with an intelligently designed training program which was not designed to evoke muscle pain, may have contributed to the lack of significant changes. This may explain the lower-than-expected pain scores and overall high wellbeing scores reported throughout the study which did not change significantly within or between groups throughout the study. Therefore, to test the effectiveness of PEA on pain/wellbeing in athletes, future studies should consider exploring the use of PEA during functional overreaching cycles, when athletes may experience significant stress and pain associated with their training.

Finally, there were no significant differences between groups or changes over time observed for sleep scores within this study. Rao et al. [26] found that eight weeks of PEA supplementation (350 mg PEA) could significantly reduce sleep latency in participants with disturbed sleep patterns. Similarly, Evangelista et al. [27] found that 1200 mg of PEA prior to bed improved sleep scores when compared to the control group in those that underwent carpal tunnel surgery (↑6.3 vs ↓3.7.). However, in this current study there was no significant effect of PEA on PSQI scores. A possible reason for the difference in this study could be the dosage used i.e. whilst our participants took 300 mg of PEA/day, they were instructed to split the dose over the day resulting in 150 mg pre-bed. Additionally, unlike previous studies [26, 27], we did not recruit participants with sleep impairments. The subjective sleep scores indicated that a high number of participants (78% and 89%) within this current study scored “poor” for sleep quality based on PSQI scores that were > 5. Therefore, future studies should consider a dose response protocol using participants with sleeping problems, or in athletes who have trouble sleeping prior to major events to assess the effectiveness of Levagen + more fully on sleep quality in an active/athletic population.

A key strength of this study was the inclusion of the double-blinded experimental design in addition to the relatively high participant number (compared to similar studies). We also included males and females in the cohort. Additionally, to enhance the accuracy of the body composition assessments we standardized and measured hydration status. The study was also comprehensive in that we measured a variety of ancillary variables (e.g., sleep, physical activity, food intake, hydration status). However, the study did include some limitations. Firstly, the participants were not a homogenous group. Although all participants that were included were at least 6-months untrained, recreationally active healthy adults, there were individuals from a variety of sporting backgrounds (e.g., endurance and team sports) with different prior experiences in resistance training. Secondly, whilst we standardized peri-workout nutrition and we monitored food intake throughout the study, we did not standardize food intake at any other meals, other than those we provided the participants around workouts and testing. Lastly, the exclusion of participants with experience in resistance training may limit the generalization of our findings to non-strength trained populations.

## Conclusions

This study is the first of its kind to evaluate the effectiveness of PEA supplementation in the context of an ecologically valid strength training protocol designed to improve skeletal muscle hypertrophy, physical strength and power in young, healthy, active males and females. We have demonstrated that a daily dose of 300 mg PEA provided during an 8-week strength training protocol does not interfere with most strength training adaptations. Furthermore, we provide evidence that PEA supplementation during strength training may improve lower body power.

### Supplementary Information

Supplementary Material 1

## Data Availability

The datasets used and/or analyzed during the current study are available from the corresponding author upon reasonable request.

## Abbreviations

1-RM: 1-Repetition maximum
AMRAP: As many reps as possible
AU: Arbitrary units
BMI: Body mass index
Cals: Calories
COX-2: Cyclooxygenase-2
DOMS: Delayed onset muscle soreness
DXA: Dual-energy X-ray absorptiometry
IMTP: Isometric mid-thigh pull
ITT: Intention to treat
MVPA: Moderate to vigorous physical activity
N: Newtons
NSAIDs: Non-steroidal anti-inflammatory drugs
OTC: Over the counter
PAEE: Physical activity energy expenditure
PEA: Palmitoylethanolamide
PLA: Placebo
PSQI: Pittsburgh sleep quality index
PP: Per protocol
pQCT: Peripheral quantitative computed tomography
SOL: Sleep onset latency
SRSS: Short recovery and stress scale
SSS: Short stress scale
SMI: Skeletal muscle index
TAC: Total activity count
W: Watts
